# Human-induced pluripotent stem cell-derived neural stem/progenitor cell *ex vivo* gene therapy with synaptic organizer CPTX for spinal cord injury

**DOI:** 10.1016/j.stemcr.2024.01.007

**Published:** 2024-02-15

**Authors:** Yusuke Saijo, Narihito Nagoshi, Momotaro Kawai, Takahiro Kitagawa, Yu Suematsu, Masahiro Ozaki, Munehisa Shinozaki, Jun Kohyama, Shinsuke Shibata, Kosei Takeuchi, Masaya Nakamura, Michisuke Yuzaki, Hideyuki Okano

**Affiliations:** 1 Department of Orthopaedic Surgery, Keio University School of Medicine, 35 Shinanomachi, Shinjuku-ku, Tokyo 160-8582, Japan; 2 Department of Physiology, Keio University School of Medicine, 35 Shinanomachi, Shinjuku-ku, Tokyo 160-8582, Japan; 3 Division of Microscopic Anatomy, Graduate School of Medical and Dental Science, Niigata University, 1-757 Asahimachi-dori, Chuo-ku, Niigata City, Niigata 951-8510, Japan; 4 Department of Medical Cell Biology, Aichi Medical University School of Medicine, 1-1 Yazago-Karimata, Nagakute, Aichi 430-1195, Japan

**Keywords:** spinal cord injury, SCI, cell transplantation, hiPSCs, ex vivo gene therapy, synapse organizer, synapse formation, CPTX

## Abstract

The transplantation of neural stem/progenitor cells (NS/PCs) derived from human induced pluripotent stem cells (hiPSCs) has shown promise in spinal cord injury (SCI) model animals. Establishing a functional synaptic connection between the transplanted and host neurons is crucial for motor function recovery. To boost therapeutic outcomes, we developed an *ex vivo* gene therapy aimed at promoting synapse formation by expressing the synthetic excitatory synapse organizer CPTX in hiPSC-NS/PCs. Using an immunocompromised transgenic rat model of SCI, we evaluated the effects of transplanting CPTX-expressing hiPSC-NS/PCs using histological and functional analyses. Our findings revealed a significant increase in excitatory synapse formation at the transplantation site. Retrograde monosynaptic tracing indicated extensive integration of transplanted neurons into the surrounding neuronal tracts facilitated by CPTX. Consequently, locomotion and spinal cord conduction significantly improved. Thus, *ex vivo* gene therapy targeting synapse formation holds promise for future clinical applications and offers potential benefits to individuals with SCI.

## Highlights

• CPTX-NS/PCs transplantation enhanced synapse formation and maturation
• CPTX-NS/PCs facilitated integration into the upper neural tract
• Enhanced synaptogenesis around the graft site led to improved spinal conductivity and gait

## Teaser

Saijo et al. provided a novel gene therapy for spinal cord injury using hiPSC-NS/PCs. By expressing the synaptic organizer CPTX from transplanted cells, they promoted synaptogenesis around the transplanted area and revealed functional recovery beyond conventional cell therapy. This approach is expected to be a promising therapeutic strategy for spinal cord injury.

## Introduction

Spinal cord injury (SCI) causes tissue damage and inflammation, resulting in the irreversible loss of neural function and permanent impairments below the injury site. Various treatment strategies have been used to reconstruct injured spinal cords. We have successfully demonstrated the efficacy of transplanting human induced pluripotent stem cell-derived neural stem/progenitor cells (hiPSC-NS/PCs) into the injured sites during the subacute phase of SCI in animal models (Nori et al., 2011). These studies have shown motor functional recovery and positive histological outcomes following the transplantation of hiPSC-NS/PCs. This procedure is being translated into clinical trials involving human subjects (Sugai et al., 2021). However, the functional recovery obtained by hiPSC-NS/PCs transplantation alone is still limited.

To further enhance the recovery, we plan to combine a novel treatment approach with conventional hiPSC-NS/PCs transplantation therapy to overcome these challenges, including *ex vivo* gene therapy (Okano, 2022a, 2022b). When considering a new therapeutic strategy, it is necessary to re-evaluate the recovery mechanisms of cell transplantation therapy for SCI (Assinck et al., 2017). One pivotal mechanism is the reconstruction of neural circuits through synaptic formation. Recent advancements in genetic technology have provided direct evidence of the connection between host and graft neurons (Ceto et al., 2020; Kitagawa et al., 2022). Intriguingly, Kawai et al. (2021) used the designer receptors exclusively activated by designer drugs system to continuously stimulate transplanted cells for 6 weeks, resulting in increased synaptic gene expression and improved motor function compared to conventional transplantation procedures. These studies demonstrated the significance of the robust connections between the host and transplanted neurons in functional motor recovery.

During development, specific neurons interconnect to form neuronal circuits through the action of various synaptic organizers. Among these, cerebellin-1 (Cbln1) (Matsuda et al., 2010; Uemura et al., 2010) and neuronal pentraxin-1 (NP1) (Xu et al., 2003; Sia et al., 2007) are unique because they are secreted from neurons in an activity-dependent manner and rapidly induce synapse formation even in mature neuronal circuits (Yuzaki, 2018). For example, injecting Cbln1 into the adult cerebellum has been demonstrated to induce rapid and potent synapse formation (Ito-Ishida et al., 2008). Although the action of Cbln1 is mainly restricted to the cerebellum, a recently developed synthetic synaptic organizer, CPTX, a chimeric protein composed of Cbln1 and NP1, can induce extrinsic synaptogenesis in many neuronal circuits (Suzuki et al., 2020). This is achieved by simultaneously binding to neurexins (NRXs) with splice site 4 (NRX-SS4) expressed in the presynaptic region and AMPA-type glutamate receptors (AMPARs) in the postsynaptic membrane. It is interesting that reanalysis of RNA sequencing data from hiPSC-NS/PSs transplanted into the spinal cord revealed that NRXs and AMPARs are expressed several weeks after transplantation (Nori et al., 2015). Therefore, we hypothesized that CPTX, continuously expressed in transplanted cells, is secreted and promotes synapse formation between the host neural circuit and the transplanted cells.

In the present study, we assessed the effects of transplanting CPTX-expressing hiPSC-NS/PCs through histological, functional, and electrophysiological analyses. Our findings reveal that the combination therapy with CPTX led to an increase in excitatory synaptic inputs on the transplanted neurons and improvements in locomotion and spinal cord conduction compared to the conventional transplantation method. Consequently, *ex vivo* gene therapy targeting synapse formation demonstrates promise for future clinical applications, presenting potential benefits for individuals with SCI.

## Results

### Establishment of hiPSC-NS/PCs expressing CPTX via lentiviral infection

**Figure 1:**
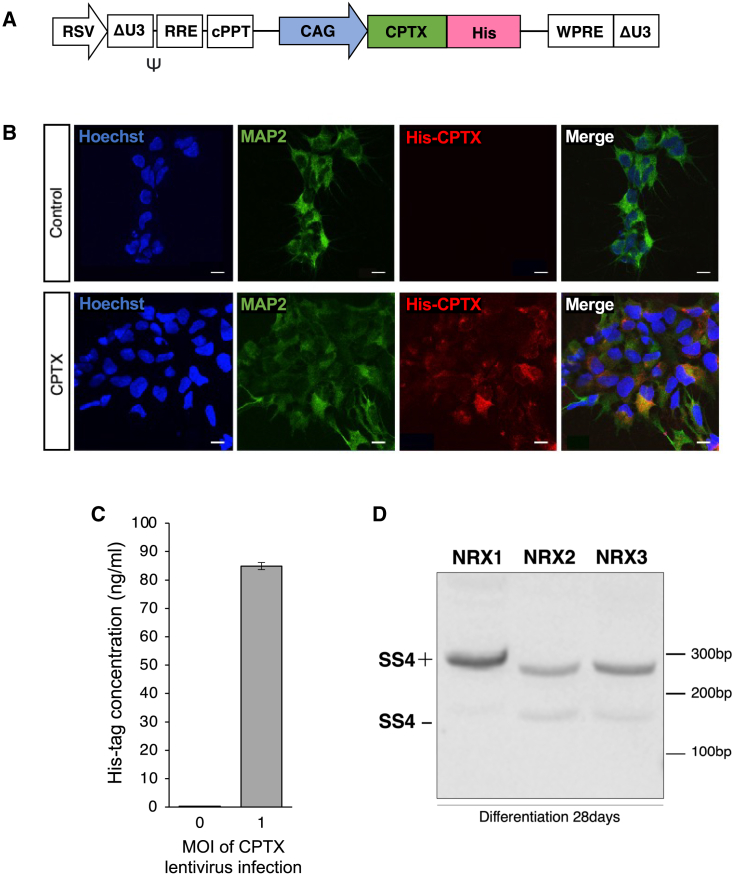
Establishment of CPTX-expressing hiPSC-NS/PCs; exocrine functional assessment and confirmation of expression of CPTX binding site from hiPSC-NS/PCs (A) Schematic illustration of the lentiviral vectors pLV-CAG-CPTX-His, which contain the CPTX gene under the control of the CAG promoter. (B) Representative image of immunostaining for MAP2 and His-tag with Hoechst from the control group (upper) and CPTX group (lower) at 7 days postdifferentiation. Scale bars: 10 μm. (C) Quantitative His-tag ELISA analysis of supernatants collected on day 3 in differentiation medium for His-tag concentration after lentiviral infection compared to without infection (MOI 0) as a negative control (n = 4 wells each independent experiments). (D) PCR reactions of the NRX SS4 gene for each subtype from hiPSC-NS/PCs at differentiation day 28. PCR products were separated by electrophoresis on 8% polyacrylamide gel.

We generated hiPSC-NS/PCs expressing CPTX (CPTX-NS/PCs) using a lentiviral vector. The *CPTX* gene was inserted into the lentivirus vector and designed to be expressed under the control of the ubiquitous CAG promoter (Miyazaki et al., 1989). In addition, a His-tag was appended to the *CPTX* genes at its C terminus, denoted as CAG-CPTX-His (Figure 1A). We verified the expression of CPTX in hiPSC-NS/PCs through immunocytochemistry. The hiPSC-NS/PCs were transduced with a lentivirus carrying the CPTX gene and then cultured in a differentiation medium for 7 days. Expression of His-tagged CPTX was confirmed in these cells, whereas the control hiPSC-NS/PCs (control-NS/PCs), which were not subjected to viral treatment, displayed no expression (Figure 1B). Subsequently, we analyzed the lactate dehydrogenase (LDH) release and the number of surviving cells following the third day of CPTX-NS/PC differentiation to evaluate the safe dose of lentivirus administration. The number of surviving cells significantly decreased when the MOI was two or higher (Figures S1A and S1B). Based on these results and considering cellular toxicity, we set a lentivirus administration dose with minimal toxicity. We analyzed whether CPTX was secreted from hiPSC-NS/PCs using a His-tag ELISA detection kit. We cultured hiPSC-NS/PCs in the differentiation medium for 3 days and used the culture supernatant to detect the His-tags. Compared with the control, the infected group showed the presence of a His-tag in the supernatant (Figure 1C). To promote synaptic formation around transplanted cells, it is crucial to have binding sites for CPTX, NRX, and AMPAR, expressed on the transplanted cells. Although reanalysis of RNA sequencing data indicated that NRXs and AMPARs are expressed in hiPSC-NS/PSs transplanted into the spinal cord several weeks after transplantation (Nori et al., 2015), it was unclear whether NRX isoforms contained a 30-residue SS4 insert, a binding site for CPTX (Matsuda et al., 2010; Uemura et al., 2010). Thus, we prepared cDNAs from hiPSC-NS/PCs cultured for 28 days and examined the presence of the SS4 insert for each NRX isoform using primers flanking the SS4 region. The results showed the presence of the SS4 insert in each NRX subtype (Figure 1D). Thus, CPTX, expressed and secreted from transplanted cells through the lentiviral vector, is expected to promote synaptogenesis.

### Transplanted CPTX-NS/PCs exhibited engraftment and long-term secretion

**Figure 2:**
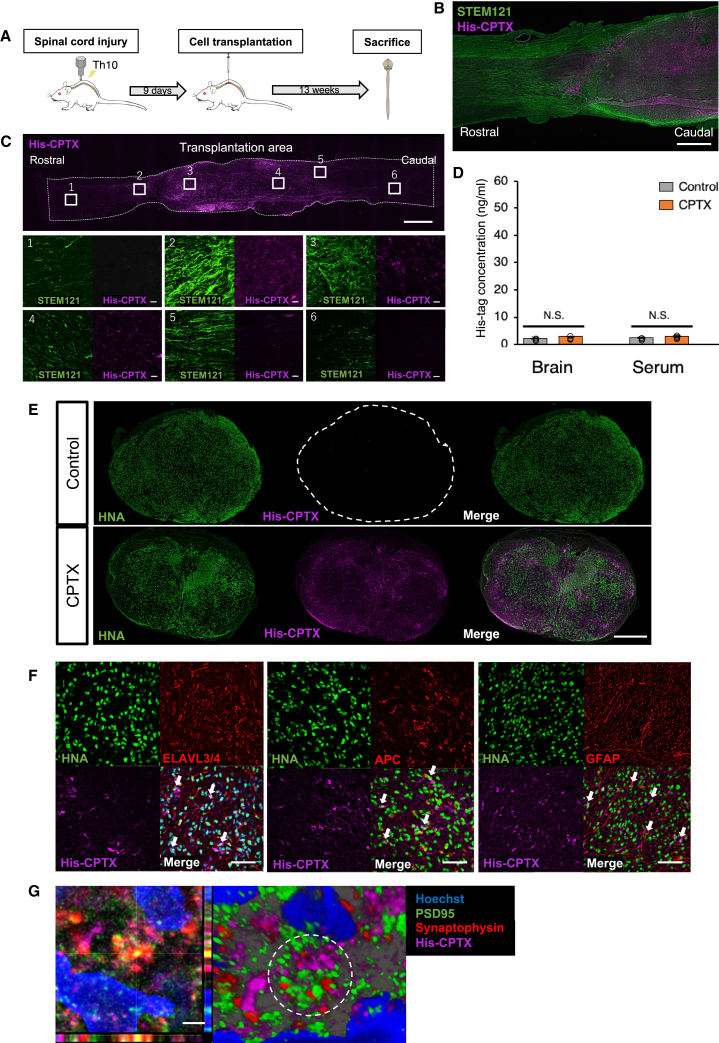
Histological appearance of a spinal cord transplanted with CPTX-NS/PCs (A) Schematic illustration of the time schedule of the *in vivo* experiments. (B) Representative image of a sagittal section stained for STEM121 (human-specific cytoplasm marker) and His-tag at 13 weeks after CPTX-expression NS/PCs transplantation. Scale bar: 1 mm. (C) Representative images of sagittal section for distribution of His-tag^+^ area with STEM121 staining, lower images (1–6): (1) +6 mm rostral area from the transplanted region, (2) +2 mm rostral area, (3) and (4) transplanted site, (5) +2 mm caudal area, (6) +6 mm caudal area. Scale bars: upper image: 1 mm, lower image: 10 μm. (D) Quantitative ELISA analysis using anti-His-tag antibody from brain and venous serum in the control group (without lentivirus administration) and CPTX group at 13 weeks after transplantation (n = 4 each, brain p = 0.28, serum p = 0.26). Statistical analysis was performed using the Mann-Whitney *U* test. (E) Representative images of axial sections showing the distribution of His-tag and HNA^+^ cells. (Upper) control group (lower) CPTX group. Scale bar: 1 mm. (F) Representative images of His-tag-expressing cells merged with ELAVL3/4 (a neuronal marker), APC (an oligodendrocyte marker), GFAP (an astrocyte marker), and HNA. His-tag, ELAVL3/4 or APC or GFAP, HNA, and triple-positive cells (white arrows). Scale bars: 50 μm. (G) Representative images of synapse formation using human-specific synaptophysin (presynaptic marker) and PSD95 (postsynaptic marker), and accumulation of His-tags in the synaptic cleft (right; dotted circle). The right image showed 3-dimensional reconstruction of the left image. Scale bar: 10 μm. Values are the mean ± SEM. N.S., not significant; ^∗^p < 0.05, ^∗∗^p < 0.01.

We created a thoracic SCI model using an Infinite Horizon impactor in immunocompromised rats. Nine days after SCI (corresponding to the subacute phase), we transplanted CPTX-NS/PCs to evaluate the spinal cord tissue after a 13-week follow-up (Figure 2A). Histological evaluation was compared in three groups: no cell transplantation group (PBS group), control-NS/CPs transplantation group (control group), and CPTX-NS/PCs transplantation group (CPTX group). In the spinal cord tissue, at the endpoint, the PBS group exhibited significant cavities within the center of the injury and atrophy of the spinal cord. However, these conditions improved in the control and CPTX groups (Figure S2A). In addition, we confirmed the engraftment of the transplanted cells and sustained expression of CPTX at the transplantation site (Figure 2B). We evaluated the distribution of CPTX around the epicenter. The distribution of CPTX was limited to the transplantation site in the sagittal spinal cord slices. CPTX expression was almost absent when the distance from the transplantation site exceeded ∼6 mm in the rostral-caudal direction (Figure 2C). To investigate potential issues related to the long-term sustained expression of CPTX, including abnormal synapse formation in normal tissues through blood circulation, we collected brain tissue and venous serum and tested whether the His-tag could be detected using ELISA. The results showed that almost undetectable level of His-tag was observed in the brain or serum (brain: 2.31 ± 0.43 ng/mL vs. 2.89 ± 0.21 ng/mL, p = 0.28, serum: 2.45 ± 0.20 ng/mL vs. 3.04 ± 0.17 ng/mL, p = 0.26) (Figure 2D). This suggests that abnormal synapse formation was almost negligible and unlikely to occur at locations distant from the transplantation site. By combining the results of the ELISA and tissue imaging, we concluded that CPTX was localized at the transplantation site and likely exerted its effects around the injured area without diffusion to other regions. In the horizontal section of the spinal cord of the CPTX group, His-tag^+^ cells were widely expressed at the transplantation site compared with the control group (Figure 2E). The transplanted His-tag^+^ cells merged with ELAVL3/4^+^ neurons, adenomatous polyposis coli (APC)^+^ oligodendrocytes, and glial fibrillary acidic protein (GFAP)^+^ astrocytes, indicating their differentiation into the three major types of neural cells (Figure 2F). In the vicinity of His-tag-expressing cells, we observed an accumulation of the secreted His-tag in the synaptic cleft (Figure 2G).

### The CPTX-NS/PCs differentiated into neural cells in the injured spinal cord

**Figure 3:**
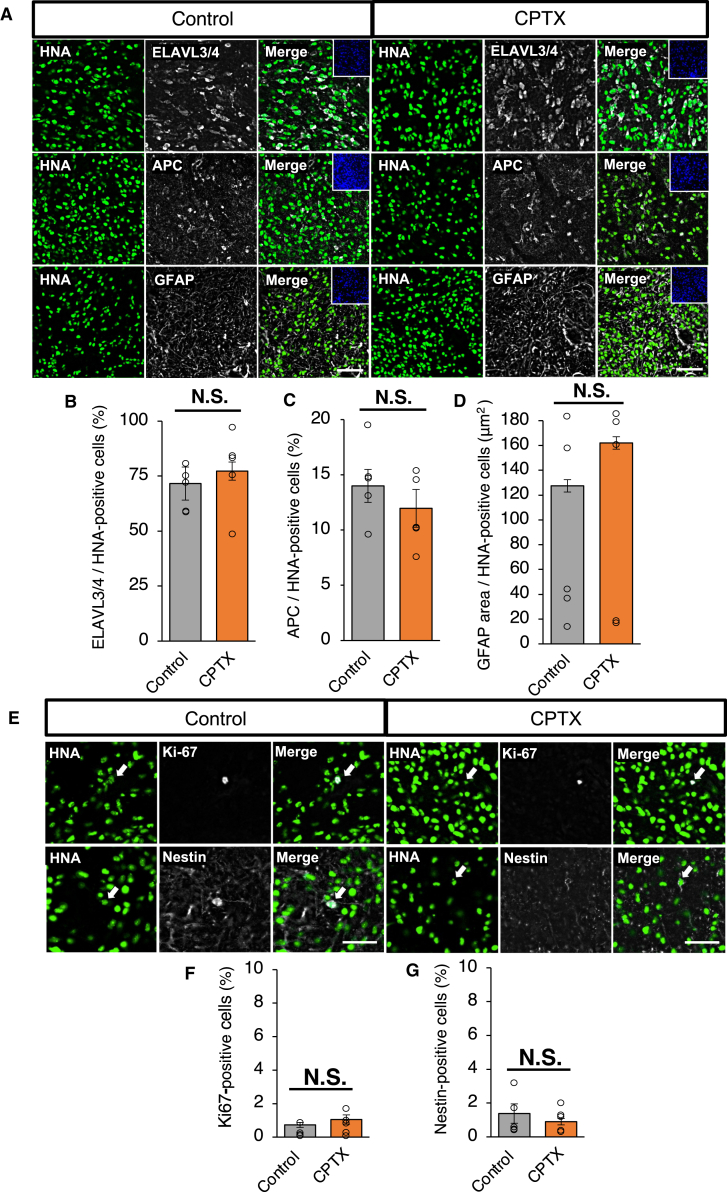
The differentiation and immature cell profiles of CPTX-NS/PCs and control-NS/PCs (A) Representative images of HNA^+^ cells expressing ELAVL3/4, APC, and GFAP in the control group (left) and CPTX group (right). Scale bars: 50 μm. (B and C) Proportions of ELAVL3/4 and APC cells among the HNA^+^ cells (n = 5 each, ELAVL3/4 p = 0.53, APC p = 0.29). (D) Quantification of GFAP^+^ area per HNA transplanted cells (n = 5 each, p = 0.65). (E) Representative images of immature graft cells in the control group (left) and CPTX group (right). HNA^+^ engrafted cells merged with Nestin (immature cell) and Ki67 (immature cell) in each transplanted group. Scale bars: 20 μm. (F and G) Quantification of immature cells in engrafted cells (n = 5 each, Nestin p = 0.45, Ki67 p = 0.34). Values are the mean ± SEM. ^∗^p < 0.05; ^∗∗^p < 0.01. Statistical analysis was performed using the Mann-Whitney *U* test in (B)–(D), (F), and (G).

CPTX-NS/PCs (CPTX group) or control-NS/PCs (control group) were transplanted into rats with subacute SCI. Thirteen weeks posttransplantation, we performed a histological analysis to evaluate the differentiation profile. CPTX-NS/PCs differentiated into ELAVL3/4^+^ neurons, APC^+^ oligodendrocytes, and GFAP^+^ astrocytes. We evaluated the differentiation profiles of the engrafted cells by double staining with human nuclear antigen (HNA) and compared them with those of the control group. The results showed no significant differences between the two groups in terms of the percentages of ELAVL3/4^+^ neurons (71.4% ± 7.5% vs. 77.3% ± 5.0%, p = 0.53), APC^+^ oligodendrocytes (14.5% ± 1.5% vs. 12.0% ± 1.7%, p = 0.29), and GFAP^+^ astrocytes (127.5 ± 53.9 μm^2^ vs. 162.1 ± 51.5 μm^2^, p = 0.65) (Figures 3A–3D). Furthermore, to evaluate potential tumor formation by the transplanted cells, we quantified the number of immature cells compared to the control group. We assessed the cell proliferation marker Ki67 (0.7% ± 0.2% vs. 1.1% ± 0.3%, p = 0.34) and the neural stem cell marker Nestin (1.4% ± 0.6% vs. 0.9% ± 0.2%, p = 0.45) (Figures 3E–3G), and there were no significant differences between the two groups. These results suggested that lentivirus administration or CPTX expression did not affect the differentiation capacity in neural cell lineages or the proliferation of immature cells, indicating no tumor formation.

### CPTX promotes synapse formation around the epicenter

**Figure 4:**
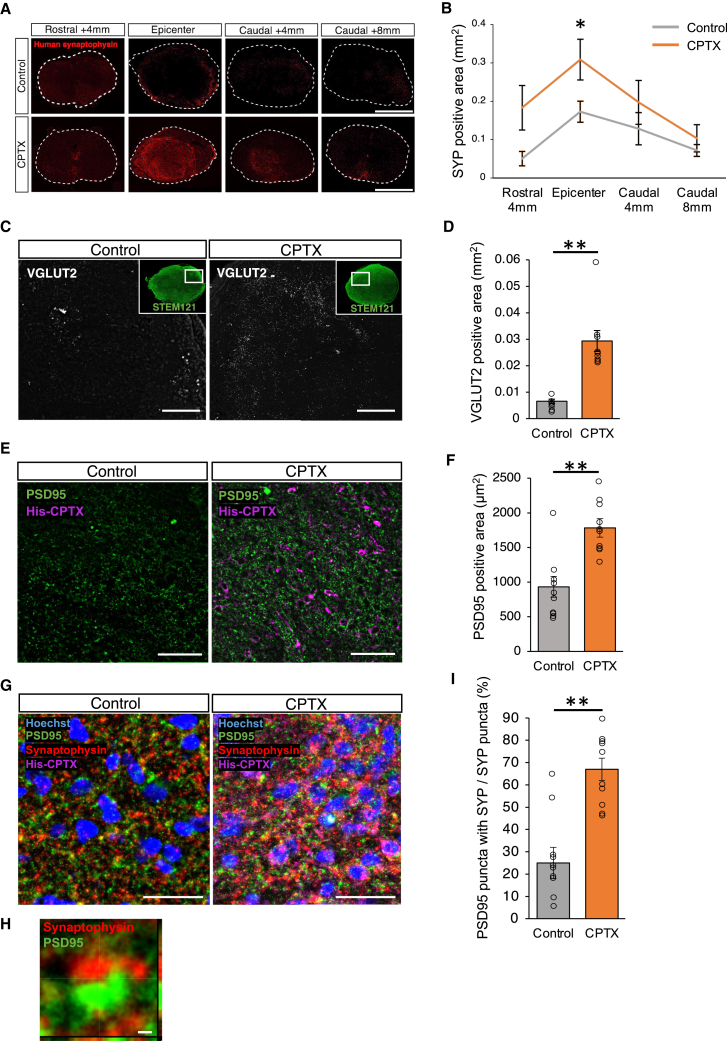
CPTX-NS/PCs transplantation promotes synapse formation (A) Representative images of the human-specific synaptophysin^+^ area at the epicenter, at site 4 mm rostral and caudal, and at site 8 mm caudal from the NS/PCs-transplanted rats’ spinal cords at 14 weeks after SCI, with or without lentivirus-induced CPTX gene. Scale bars: 1 mm. (B) Quantitative analysis of the human-specific synaptophysin (SYP)^+^ area in the axial section (control group n = 7, CPTX group n = 8), (rostral 4 mm p = 0.28, epicenter p = 0.045, caudal 4 mm p = 0.35, caudal 8 mm p = 0.72). (C) Representative images of VGlut2^+^ (excitatory presynaptic marker) area at the center of transplantation. Scale bars: 100 μm. (D) Quantitative analysis of VGlut2^+^ area in axial section (control group n = 7, CPTX group n = 9, p = 0.00006). (E) Representative images of PSD95^+^ area around CPTX-expressing cells in axial section. Scale bars: 100 μm. (F) Quantitative analysis of PSD95^+^ is in the axial section (n = 10 slices each from 5 rats, p = 0.0005). (G and H) Representative images of axial section stained for PSD95, SYP, and His-tag with Hoechst. Magnified image of contact with SYP (presynaptic marker) and PSD95 (postsynaptic marker). Scale bars: 50 μm (G) and 10 μm (H). (I) Quantitative analysis of the fraction of PSD95^+^/SYP^+^-double-positive puncta (n = 10 slices each from 5 rats, p = 0.0001). Values are the mean ± SEM. ^∗^p < 0.05; ^∗∗^p < 0.01. Statistical analyses were performed using the Mann-Whitney *U* test in (B), (D), (F), and (I).

Immunohistochemical evaluation using various synaptic markers was performed to demonstrate the effects of diffused CPTX on the interactions between the transplanted and host cells. To analyze synaptic maturation derived from the transplanted cells, we quantitatively evaluated the area of human-specific synaptophysin, a presynaptic marker, in the injured vicinity compared with that in the control group. The results showed no significant differences between the control and CPTX groups in the rostral 4 mm (0.051 ± 0.019 mm^2^ vs. 0.18 ± 0.058 mm^2^, p = 0.28), caudal 4 mm (0.128 ± 0.042 mm^2^ vs. 0.197 ± 0.057 mm^2^, p = 0.35), and caudal 8 mm (0.071 ± 0.015 mm^2^ vs. 0.103 ± 0.035 mm^2^, p = 0.72). However, a significant increase in the synaptophysin area was observed in the epicenter compared to the control group (0.308 ± 0.053 mm^2^ vs. 0.173 ± 0.027 mm^2^, p = 0.045) (Figures 4A and 4B). CPTX has been shown to induce synaptogenesis by forming the NRX-CPTX-AMPAR tripartite complex between AMPAR- and NRX(SS4)-expressing cells (Suzuki et al., 2020). Therefore, although the precise region (axon, dendrite, or soma) from which CPTX is secreted and the extent of its diffusion are not fully understood, the increase in synaptophysin suggests the accumulation of axon terminals of transplanted cells due to CPTX. To evaluate the overall excitatory activation in the transplantation center after excitatory synaptic formation induced by CPTX, immunostaining was performed using vesicular glutamate transporter 2 (VGlut2), an excitatory vesicular protein. The VGlut2^+^ areas were quantitatively evaluated and compared with those of the control group at the transplantation center. The results showed that VGlut2 increased significantly in the CPTX group compared to the control group (0.029 ± 0.003 mm^2^ vs. 0.0065 ± 0.007 mm^2^, p = 0.00006) (Figures 4C and 4D). We quantitatively evaluated the area of postsynaptic density 95 (PSD95), a postsynaptic scaffolding protein, in the transplantation center. As a result, we observed a significant increase in the area of PSD95 in the CPTX group (1,782.9 ± 133.9 μm^2^ vs. 931.6 ± 150.8 μm^2^, p = 0.00005) (Figures 4E and 4F). However, we also analyzed the inhibitory synaptic marker, vesicular GABA transporter (VGAT). The VGAT^+^ area did not show any significant difference between both groups (0.019 ± 0.003 mm^2^ vs. 0.031 ± 0.007 μm^2^, p = 0.27) (Figures S3A and S3B). Synaptic formation was evaluated using antibodies against human-specific synaptophysin and PSD95. Synaptophysin/PSD95 double-positive puncta was identified. The CPTX group showed a significant increase in double-positive puncta (66.8% ± 6.9% vs. 25.5% ± 4.9%, p = 0.00001) (Figures 4G–4I). These results demonstrate that transplantation of CPTX-NS/PCs promotes synapse formation in transplanted neuronal cells and further increases excitatory signaling in the transplantation center.

### CPTX increased the synaptic connection between the transplanted cells and upper neurons

**Figure 5:**
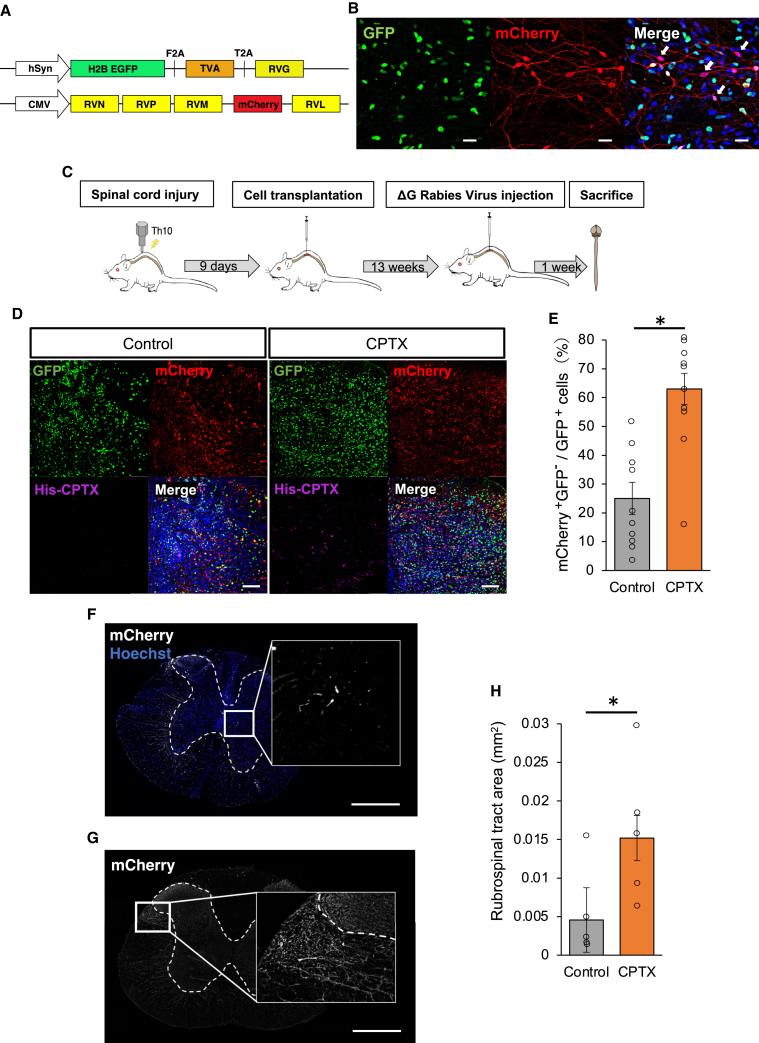
CPTX enhanced the synaptic connection between upper neural tract and graft cells, which was visualized by the retrograde monosynaptic tracing (A) Schematic illustration of GFP-TVA-glycoprotein gene construction of lentivirus vector (upper), and G-deleted rabies virus gene construction labeled with mCherry (lower). (B) Representative images of differentiated cells stained for GFP and mCherry at 4 weeks of differentiation *in vitro*. Scale bars: 10 μm. (C) Schematic illustration of the time schedule of the *in vivo* G-deleted (ΔG) rabies virus tracing experiments. (D) Representative images of engrafted cells stained for GFP, mCherry, and His-tag in the control group (left) and CPTX group (right) at the epicenter. Scale bars: 50 μm. (E) Quantitative analysis of the fraction of mCherry^+^/GFP^−^ cells. Corrected for the total number of GFP^+^ cells (n = 10 slices each from 5 rats, p = 0.001). (F) Representative images of PNs stained for mCherry at the cervical spinal cord. Scale bar: 1 mm. (G) Representative images of RST stained for mCherry at the cervical spinal cord. Scale bar: 1 mm. (H) Quantitative analysis of the bilateral RST area stained for mCherry at the cervical spinal cord (n = 5 each, p = 0.016). Values are the mean ± SEM. ^∗^p < 0.05; ^∗∗^p < 0.01. Statistical analyses were performed using the Mann-Whitney *U* test in (E) and (H).

Knowledge is still limited about the specific neural circuits in the host that are connected to transplanted cells. We are focused on understanding the origin of the upper neurons that directly connect to the transplanted cells, and whether the expression of CPTX in these transplanted cells enhances their connectivity with upper neurons. To investigate these aspects, we performed tracing experiments using glycoprotein (G)-deleted rabies virus. G-deleted rabies virus tracing experiments have been used to uncover these neural networks (Adler et al., 2017). By using retrograde monosynaptic tracing, connections with superior neural tracts can be visualized, thereby providing valuable insights into connectivity patterns. By genetically manipulating the transplanted cells to express TVA, a cognate receptor for the envelope EnvA, and glycoprotein of the rabies virus, the G-deleted rabies virus can selectively infect the targeted cells. The TVA and glycoproteins were labeled with GFP, whereas the G-deleted rabies virus was labeled with mCherry (Figure 5A). *In vitro* experiments revealed the presence of mCherry^+^/GFP^−^ cells in cultured neurons, indicating synaptic transmission of the virus (Figure 5B). hiPSC-NS/PCs were infected with lentiviral vectors expressing TVA and glycoprotein and transplanted into the injury site during the subacute phase. After 12 weeks, the G-deleted rabies virus was injected (Figure 5C). The CPTX group received an additional infection with a lentivirus carrying the CPTX gene to hiPSC-NS/PCs one day after the initial infection with lentiviral vectors expressing TVA and glycoprotein. Our initial focus was to assess the infection efficiency of cells expressing the TVA receptor and G protein in the transplant center. Immunohistochemical staining images revealed the presence of GFP^+^ cells in the transplant center of both groups. CPTX was also expressed, indicating successful dual infection with the lentivirus (Figure 5D). At the transplantation site, mCherry^+^/GFP^−^ neurons were identified, indicating that these neurons could be either host-derived neurons or transplanted neurons that did not express TVA and G protein. It was found that the number of neurons with single-positive mCherry was significantly increased in the CPTX group (24.6% ± 5.6% vs. 62.9% ± 5.4%, p = 0.001) (Figure 5E). To identify the superior neural tracts connected to the transplanted cells, the projections were confirmed using axial sections of the spinal cord at the cervical level, where transplanted neurons did not extend. Neural fibers projected to the ventral and lateral funicular regions in both groups. In addition, propriospinal interneurons (PNs) were observed in the gray matter (Figure 5F). The rubrospinal tract (RST) showed the most clearly stained area in the descending neural pathway (Figure 5G). In the CPTX group, there was a greater extent of mCherry^+^ areas observed in the RST compared to the control group (0.015 ± 0.003 mm^2^ vs. 0.0046 ± 0.004 mm^2^, p = 0.016) (Figure 5H). These results indicated that the upper neural circuits were more connected to the transplanted neural cells expressing CPTX.

### Motor function and gait analysis following transplantation of CPTX-NS/PCs

**Figure 6:**
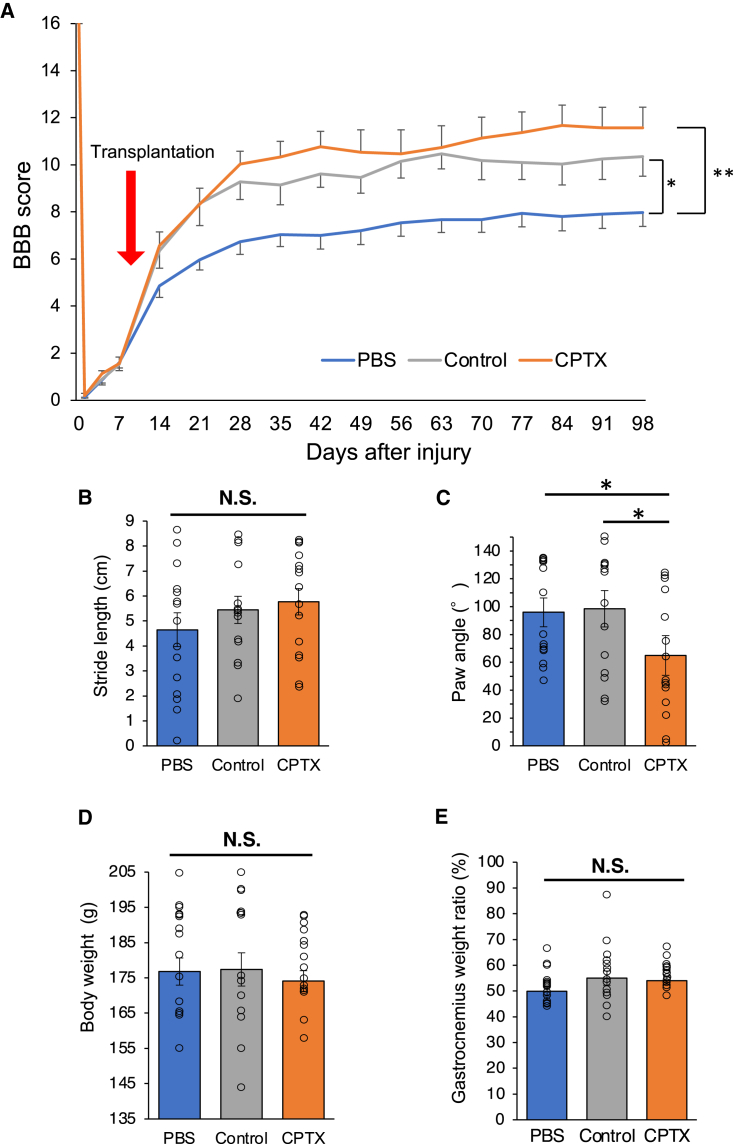
Evaluation of motor function and hindlimb muscle weight (A) Hindlimb motor function was evaluated weekly for 14 weeks after SCI by BBB score in the PBS group (noncell transplantation), the control group, and the CPTX group (PBS group, n = 15; control group, n = 14; CPTX group, n = 15; PBS vs. control, p = 0.03; PBS vs. CPTX, p = 0.002; control vs. CPTX, p = 0.58). (B) Comparison of stride lengths determined by treadmill gait analysis performed at 13 weeks after transplantation (PBS group, n = 15; control group, n = 14; CPTX group, n = 15; PBS vs. control, p = 0.45; PBS vs. CPTX, p = 0.28; control vs. CPTX, p = 0.76). (C) Comparison of paw angles determined by treadmill gait analysis performed at 13 weeks after transplantation (PBS group, n = 15; control group, n = 14; CPTX group, n = 15; PBS vs. control, p = 0.87; PBS vs. CPTX, p = 0.016; control vs. CPTX, p = 0.029). (D) Comparison of body weight at 14 weeks after SCI (PBS group, n = 15; control group, n = 14; CPTX group, n = 15; PBS vs. control, p = 0.48; PBS vs. CPTX, p = 0.52; control vs. CPTX, p = 0.46). (E) Comparison of gastrocnemius muscle rate at 14 weeks after SCI (PBS group, n = 15; control group, n = 14; CPTX group, n = 15; PBS vs. control, p = 0.099; PBS vs. CPTX, p = 0.078; control vs. CPTX, p = 0.85). Values are mean ± SEM. ^∗^p < 0.05; ^∗∗^p < 0.01. Statistical analyses were performed using the 2-way repeated-measures ANOVA with Tukey’s test (A), and the Mann-Whitney *U* test following the Kruskal-Wallis test in the treadmill gait analysis (B and C) and body and muscle weight analysis (D and E).

Regarding the assessment of motor function, the Basso, Beattie, Bresnahan (BBB) scale (Basso et al., 2006) and treadmill gait analysis were used to evaluate three groups: PBS, control, and CPTX. The CPTX and control groups showed significant improvements in BBB scores compared with the PBS group (Figure 6A) (PBS: 7.97 ± 0.58, control: 10.36 ± 0.84, CPTX: 11.57 ± 0.88, PBS vs. control, p = 0.03; PBS vs. CPTX, p = 0.002; control vs. CPTX, p = 0.58). Treadmill gait analysis was performed 13 weeks after transplantation. No significant differences were observed in stride length among the three groups (Figure 6B) (PBS: 4.7 ± 0.68 cm, control: 5.4 ± 0.54 cm, CPTX: 5.8 ± 0.53 cm, PBS vs. control, p = 0.45; PBS vs. CPTX, p = 0.28; control vs. CPTX, p = 0.76). The CPTX group showed a significant improvement in paw angle compared to the other groups (Figure 6C) (PBS: 95.9° ± 10.3°, control: 98.5° ± 13.1°, CPTX: 64.9° ± 14.2°, PBS vs. control, p = 0.87; PBS vs. CPTX, p = 0.016; control vs. CPTX, p = 0.029). During the final observation period, there were no significant differences among the groups in body weight (PBS: 176.8 ± 3.8 g, control: 177.4 ± 4.7 g, CPTX: 174.1 ± 2.92 g, PBS vs. control, p = 0.95; PBS vs. CPTX, p = 0.52; control vs. CPTX, p = 0.46) or gastrocnemius weight ratio (PBS: 0.499 ± 0.018%, control: 0.55 ± 0.028%, CPTX: 0.54 ± 0.014%, PBS vs. control, p = 0.099; PBS vs. CPTX, p = 0.078; control vs. CPTX, p = 0.85) (Figures 6D and 6E).

### Transplantation of CPTX-NS/PCs did not exacerbate allodynia and facilitated electrophysiological recovery

**Figure 7:**
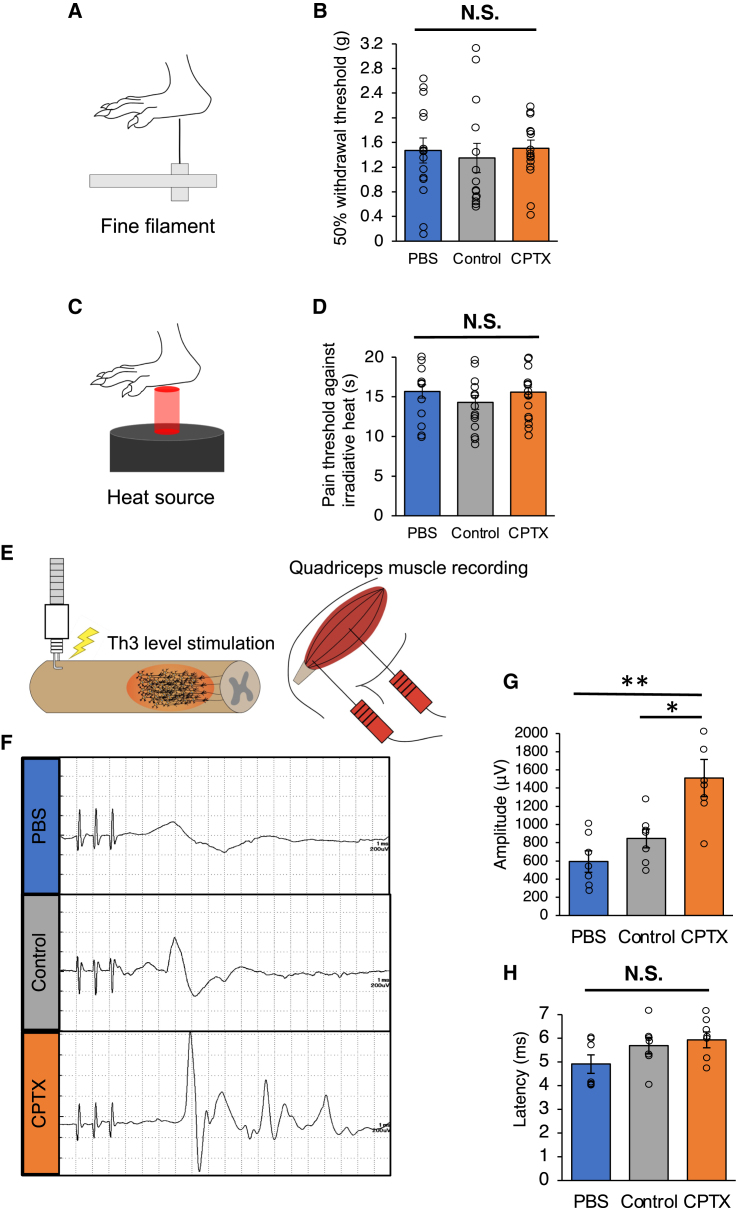
Transplantation of CPTX-NS/PCs did not aggravate allodynia and recovered electrophysiological function (A) Illustration of the von Frey test (mechanical stimulus). (B) Hind paw pain scores as determined by the von Frey test (PBS group, n = 15; control group, n = 14, CPTX group, n = 15; PBS vs. control, p = 0.49; PBS vs. CPTX, p = 0.67; control vs. CPTX, p = 0.26). (C) Illustration of Hargreaves test (heat stimulus). (D) Hind paw pain score as determined by Hargreaves test (PBS group, n = 15; control group, n = 14; CPTX group, n = 15; PBS vs. control, p = 0.22; PBS vs. CPTX, p = 0.89; control vs. CPTX, p = 0.27). (E) Illustration of the MEP experiment. (F) Representative images of MEP waves in PBS, control, and CPTX groups. (G) Quantitative analysis of MEP maximal amplitude in 3 groups (n = 7 each) (PBS vs. control, p = 0.46; PBS vs. CPTX, p = 0.001; and control vs. CPTX, p = 0.0014). (H) Quantitative analysis of MEP latency in 3 groups (n = 7 each, PBS vs. control, p = 0.3; PBS vs. CPTX, p = 0.13; control vs. CPTX, p = 0.88). Values are mean ± SEM. ^∗^p < 0.05; ^∗∗^p < 0.01. Statistical analysis was performed using the Mann-Whitney *U* test following the Kruskal-Wallis test for allodynia and MEP analysis (B, D, G, H).

Excitatory synapses containing AMPARs between primary afferent fibers and spinal dorsal horn neurons play a crucial role in sensory transmission and modulation (Li et al., 1999). Considering the potential enhancement of nociceptive pain in the sensory tract by CPTX, we assessed allodynia. Mechanical stimulation tests (von Frey filaments test, up-and-down method) (Dixon et al., 1991; Wang et al., 2018) and thermal stimulation tests (Hargreaves et al., 1988) were performed and evaluated at the observation period. Statistical analysis did not show significant differences among the three groups in either the von Frey filaments test (PBS: 1.47 ± 0.2 g, control: 1.35 ± 0.24 g, CPTX: 1.51 ± 0.13 g, PBS vs. control, p = 0.49; PBS vs. CPTX, p = 0.67; control vs. CPTX, p = 0.26) or the thermal test (PBS: 15.67 ± 0.83 s, control: 14.3 ± 0.89 s, CPTX: 15.59 ± 0.62 s, PBS vs. control, p = 0.22; PBS vs. CPTX, p = 0.89; control vs. CPTX, p = 0.27) (Figures 7A–7D). These results indicate that CPTX-NS/PCs transplantation does not have a significant effect on neuropathic pain. To gain insights into why CPTX did not affect nociceptive pain, we conducted immunohistochemical (IHC) analyses on calcitonin gene-related peptide, a presynaptic marker associated with the pain pathway (Christensen and Hulsebosch, 1997; Rogoz et al., 2014), in the lumbar dorsal horn, which is involved in the sensation of the plantar region. As with the allodynia tests, no significant differences were observed among the three groups (PBS: 0.105 ± 0.0069 mm^2^, control: 0.124 ± 0.011 mm^2^, CPTX: 0.109 ± 0.0071 mm^2^, PBS vs. control, p = 0.26; PBS vs. CPTX, p = 0.32; control vs. CPTX, p = 0.14) (Figures S4A and S4B). Since CPTX selectively induces synaptogenesis between neurons expressing NRX(SS4) and AMPARs, the lack of effect of CPTX on nociceptive pain may be attributed to the insufficient expression of NRX(SS4) and AMPAR, the receptors for CPTX, along the nociceptive pathway. Next, to clarify whether the CPTX-increased excitatory synapses are functional, we examined motor-evoked potential (MEP) using T helper 3 cell-level electrical stimulation and recording in the quadriceps femoris muscle was used for the electrophysiological evaluation of lower-limb motor function recovery following SCI (Nori et al., 2011) (Figure 7E). Monosynaptic MEPs were observed in all of the rats 13 weeks after cell transplantation, but some showed polysynaptic MEPs (Figure 7F). The amplitudes of MEPs were significantly larger in the CPTX group compared to the other groups (PBS: 592.70 ± 117.69 μV, control: 847.28 ± 103.52 μV, CPTX: 1510.72 ± 204.04 μV, PBS vs. control, p = 0.46; PBS vs. CPTX, p = 0.001; control vs. CPTX, p = 0.014) (Figure 7G). There were no significant differences in latency among the three groups (PBS: 4.91 ± 0.39 ms, control: 5.69 ± 0.35 ms, CPTX: 5.93 ± 0.33 ms, PBS vs. control, p = 0.3; PBS vs. CPTX, p = 0.13; control vs. CPTX, p = 0.88) (Figure 7H). These results suggest that CPTX expression induces functional excitatory synapses between transplanted cells and host neuronal circuits.

## Discussion

In this study, we evaluated the efficacy of transplanting hiPSC-NS/PCs transduced with CPTX gene via lentiviral transduction for the treatment of SCI. We observed robust enhancement in the formation and maturation of synapses around the transplanted cells, exceeding the outcomes of conventional transplantation. Furthermore, we promoted the integration of the transplanted neurons with host-derived superior neural tracts, leading to the reconstruction of neural circuits. These findings were reflected in the improved paw angle and spinal conduction. In addition, the lentivirus and the long-term expression of CPTX did not lead to any adverse events, such as tumor formation or worsening of allodynia, throughout our experimental procedures. Therefore, our procedure can become a valuable tool for future gene therapy.

Previous studies have highlighted the significance of synapse formation between transplanted and host cells in the injured spinal cord (Abematsu et al., 2010; Kitagawa et al., 2022). Localized neural circuitry involving the transplanted cells is known to connect with the upper descending neural tracts on the rostral side of the injury (Adler et al., 2017; Kadoya et al., 2016) and directly with motor neurons on the caudal side (Kitagawa et al., 2022; Nori et al., 2011). In our study, compared to the conventional transplantation-alone group, successful induction of the CPTX gene into hiPSC-NS/PCs robustly induced excitatory synapse formation on transplanted cells (Figure 4G). This is most likely achieved by CPTX, secreted by transplanted cells, forming the tripartite complex (NRX-CPTX-AMPAR) and recruiting axon terminals expressing NRX(SS4). Furthermore, the increased amplitude of MEPs in rats transplanted with CPTX-NS/PCs indicates that functional excitatory synapses were formed between transplanted cells and host neuronal circuits.

Although CPTX induced robust excitatory synapse formation on transplanted neurons, its effects were limited in certain behavioral assays. One potential explanation is linked to how CPTX is secreted. Unlike the administration of exogenous CPTX protein, the impact of CPTX expressed in neurons relies on its secretion patterns. If CPTX is inadequately secreted from axons, then the axons of transplanted neurons may fail to form synapses with the host neuronal circuitry. Future studies investigating the secretory patterns of CPTX may be necessary to optimize the synaptogenic potential of CPTX expressed in neurons.

We have previously reported that a single injection of CPTX protein around the injured spinal cord after SCI rapidly restores motor function by inducing the formation of collateral pathways (Suzuki et al., 2020). However, cell transplantation may be necessary to replace neurons lost due to severe damage. However, if the transplanted cells are not mature enough to express the CPTX receptors, NRXs, and AMPARs, then they are not expected to form synapses with the neural circuits of the host, even if the CPTX protein is injected with the transplanted cells. The *ex vivo* gene therapy method we developed in this study to express CPTX has the advantage of compensating for this disadvantage and promoting synapse formation of transplanted neurons at different developmental stages with host neurons. An important question for future study is how to exploit these characteristics of two treatment modalities depending on the timing and severity of different SCIs.

Significant increases were observed in proteins associated with excitatory synapses in the CPTX group (Figures 4C and 4E), suggesting that the long-term CPTX expression promotes the formation of excitatory synapses. Regarding the significance of excitatory neurons in cell therapy, a previous study reported the transplantation of excitatory neuron-enriched NS/PCs after SCI and showed functional and electrophysiological recovery (Zholudeva et al., 2018). Because excitatory neurons are known to influence gait function by directly connecting to motor neurons (Crone et al., 2008; Song et al., 2018), increased excitatory synaptic connections could have contributed to a positive effect on motor function in the present study. However, a concern remains about the occurrence of allodynia, induced by the excessive formation of excitatory synapses (Crosby et al., 2015; Scherrer et al., 2010). Our results did not reveal any significant exacerbations in the CPTX group. Taken together, promoting the formation of excitatory synapses through CPTX-NS/PCs transplantation is considered a valuable strategy from both effectiveness and safety perspectives.

Although our histological results revealed robust synaptic connections around the lesion area, we still did not fully understand how the transplanted neurons integrated into the surviving host neurons. To address these issues, we used a G-deleted rabies virus to assess the integration of grafted cells into superior neural tracts. This retrograde monosynaptic tracing technique revealed inputs from the RST to the transplanted neurons that were enhanced by CPTX induction (Figures 5F and 5G). The RST contains abundant excitatory neurons (Liang et al., 2012) and is directly connected to the motor neurons (Kuchler et al., 2002; Wild et al., 2017). This tract is strongly associated with motor function because previous studies have demonstrated functional limb impairments after the transection or ablation of the RST (Fujito and Aoki, 1995; Pettersson et al., 2000; Whishaw et al., 1998). Based on this, we believe that enhanced neural transmission from the RST to the transplanted cells contributes to the observed functional benefit. Thus, the present tracing method clarifies some of the mechanisms underlying the effectiveness of CPTX.

In CNS development, synapse formation is initially activity independent during fetal stages. As development progresses, activity-dependent synaptic refinement enhances motor learning (Parker, 2000; Südhof, 2018). CPTX promotes activity-independent synaptic formation, which is supported by histological findings. To restore motor function, activity-dependent synapse formation through locomotor training is crucial. In SCI animal models, rehabilitation improves synapse remodeling and motor function (Grau et al., 2020; Ilha et al., 2019). Furthermore, combining rehabilitation with cell transplantation enhances synaptic activity (Tashiro et al., 2016). These results suggest the potential for the synergistic effects of combining CPTX-NS/PCs transplantation with these therapies, leading to further improvements in therapeutic outcomes.

In conclusion, *ex vivo* gene therapy using hiPSC-NS/PCs with CPTX effectively enhanced synapse formation around the transplantation site and successfully integrated the transplanted neurons into the upper tract. These histological changes contributed to improved locomotion and enhanced spinal conduction. When combined with rehabilitation strategies, this approach has the potential to become a promising and valuable tool.

## Experimental procedures

### Resource availability

#### Lead contact

Narihito Nagoshi (nagoshi@2002.jukuin.keio.ac.jp).

#### Materials availability

The materials included in this study are available from the corresponding author upon reasonable request.

#### Data and code availability

The datasets generated in the present study are available from the corresponding author upon reasonable request.

### Lentiviral vector preparation

Recombinant lentiviral vector production was performed as described previously (Miyoshi et al., 1998). The details are described in the supplemental information.

### Cell culture and lentiviral transduction

We used the human umbilical cord-derived hiPSC line YZWJs513, derived from a clinical-grade human leukocyte antigen superdonor line. The detailed methods are described in the supplemental information.

### *In vitro* and *in vivo* His-tag detection ELISA analysis

hiPSC-NS/PCs infected with lentiviruses were cultured for 3 days, and His-tag concentrations were determined using a His-tag ELISA detection kit. Brain and venous serum samples from rats at 13 weeks posttransplantation were also used to measure His-tag concentrations with the same ELISA kit. The details are described in the supplemental information.

### PCR and electrophoresis for NRX SS4 detection

We examined NRX SS4 expression in cultured hiPSC-NS/PCs after 28 days. Detailed methods are described in the supplemental information.

### Cell viability and LDH release assay

Cell toxicity after lentivirus administration was assessed using Cell Counting Kit-8 and LDH release with the Cytotoxicity LDH Assay Kit. The details are described in the supplemental information.

### Animals

Adult (8-week-old) female athymic nude rats (F344/NJcl-*rnu*/*rnu*, weight = 110–180 g, CLEA Japan, Tokyo, Japan) were used for these experiments. The details are provided in the supplemental information.

### Surgical procedures

Contusive SCI was induced at the level of the tenth thoracic spinal vertebra using an Infinite Horizon impactor. Nine days after the injury, hiPSC-NS/PCs (1 × 10^6^ cells) were injected. The details are provided in the supplemental information.

### Histological analyses

Histological analyses were performed by H&E staining and IHC staining. The detailed methods and antibodies used for IHC staining are described in the supplemental information.

### G-deleted rabies virus tracing experiment

The G-deleted rabies virus was created using a method that was previously reported (Osakada and Callaway, 2013; Masaki et al., 2022). The methods for creating viruses and virus administration procedures are provided in the supplemental information.

### Quantification of staining

IHC staining of all of the sections was quantified using ImageJ software. The detailed measurement methods for each experiment have been documented in the supplemental information.

### MEP experiments

MEP experiments were performed in the PBS, control, nd CPTX groups after 98 days posttransplantation. The detailed methods are described in the supplemental information.

### Statistical analyses

The statistical analyses were performed using SPSS (version 26.0.0.0, IBM Japan, Tokyo, Japan). The detailed statistical methods are described in the supplemental information.

## Supplemental information

Document S1. Figures S1–S4 and supplemental experimental procedures

Document S2. Article plus supplemental information
