# Supplementary material for: Human-induced pluripotent stem cell-derived neural stem/progenitor cell ex vivo gene therapy with synaptic organizer CPTX for spinal cord injury
Source: Stem Cell Reports. 2024 Feb 15;19(3):383–98. doi: 10.1016/j.stemcr.2024.01.007 (PMC10937157; doi:10.1016/j.stemcr.2024.01.007)
Supplement: Document S1. Figures S1–S4 and supplemental experimental procedures [file mmc1.pdf]

Stem Cell Reports, Volume *19*

Supplemental Information

Human-induced pluripotent stem cell-derived neural stem/progenitor cell *ex vivo* gene therapy with synaptic organizer CPTX for spinal cord injury

Yusuke Saijo, Narihito Nagoshi, Momotaro Kawai, Takahiro Kitagawa, Yu Suematsu, Masahiro Ozaki, Munehisa Shinozaki, Jun Kohyama, Shinsuke Shibata, Kosei Takeuchi, Masaya Nakamura, Michisuke Yuzaki, and Hideyuki Okano

Supplemental information

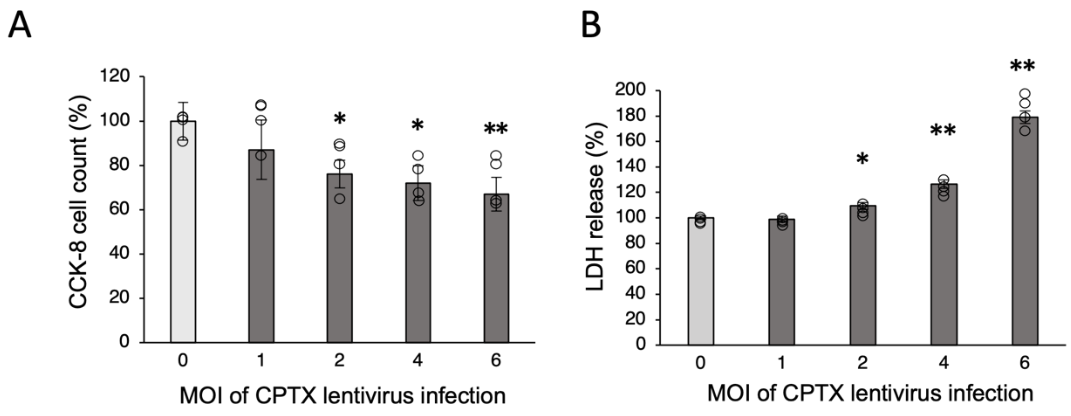

Figure S1. Evaluation of lentivirus-induced cytotoxicity and cell viability

(A) Measurement of neural cell counts using CCK-8 assays after lentiviral administration with various doses (MOI1, 2, 4, 6), compared with negative control. 100.00% [8.51%]; MOI1, 86.67% [13.43%], $p = 0.26$; MOI2, 75.75% [6.29%], $p = 0.016$; MOI4, 71.75% [7.76%], $p = 0.011$; MOI6, 66.77% [7.61%], $p = 0.005$; $n = 4$ each independent experiments.

(B) LDH release from neural cells after lentiviral administration at various doses (MOI1, 2, 4, 6), compared with the negative control. 100.00% [1.27%]; MOI1, 98.9% [2.39%], $p = 0.72$; MOI2, 109.43% [2.87%], $p = 0.024$; MOI4, 126.48% [3.22%], $p = 0.0009$; MOI6, 179.06% [4.97%], $p = 0.0005$. $n = 4$ each independent experiments.

Values are the mean ± SEM. Not significant (N.S.), $*p < 0.05$, $**p < 0.01$. Statistical analyses were performed using a Mann-Whitney U-test in (A) and (B).

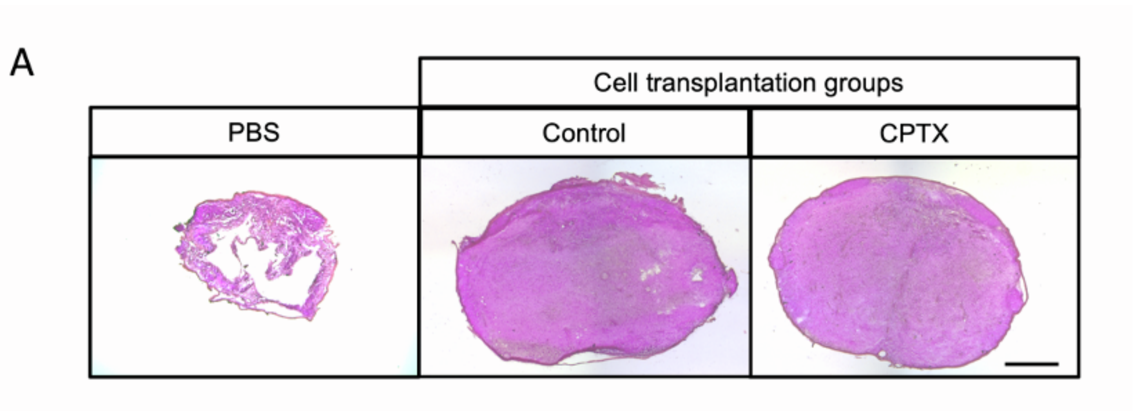

Figure S2. H&E staining of a spinal cord sections

(A) Representative images of the H&E-stained axial section at the epicenter after

14 weeks after SCI. PBS group (left), control group (middle), CPTX group (right). Scale bar 500μm.

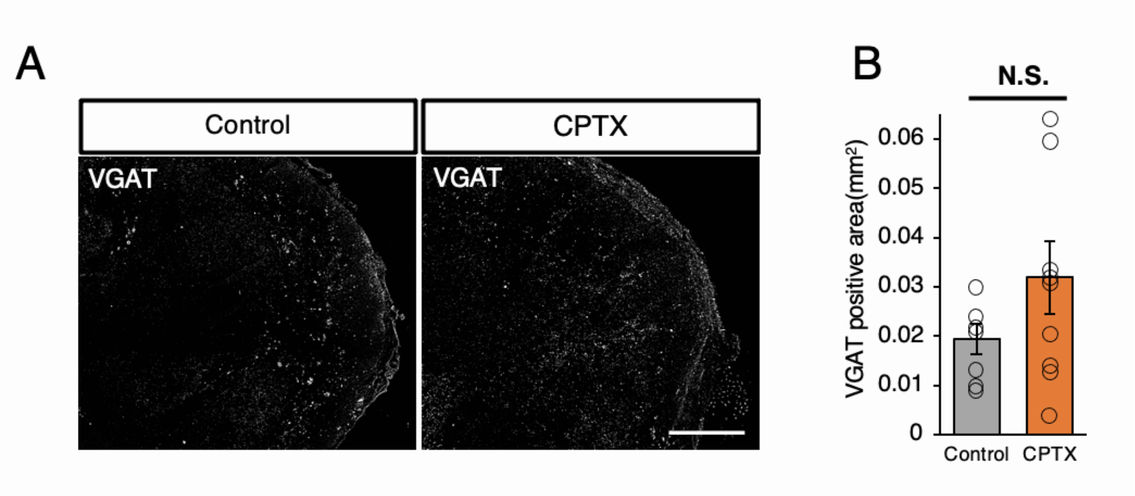

Figure S3. Immunohistochemical staining with inhibitory synaptic markers

(A) Representative images of VGAT (Inhibitory presynaptic marker)-positive area at center of transplantation. Scale bars: 100 μm.

(B) Quantitative analysis of VGAT-positive area in axial section (control group $n = 7$, CPTX group $n = 9$, $p = 0.27$).

Values are the mean ± SEM. Not significant (N.S.), $*p < 0.05$, $**p < 0.01$. Statistical analyses were performed using a Mann-Whitney U-test.

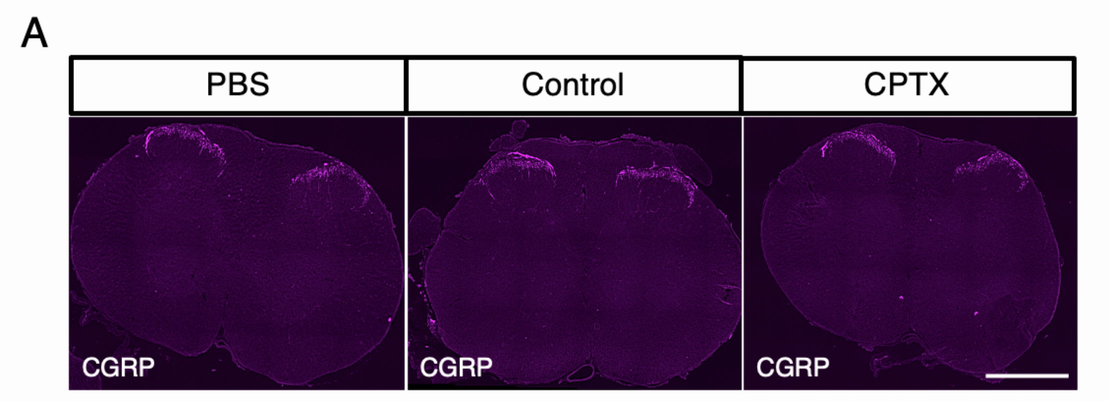

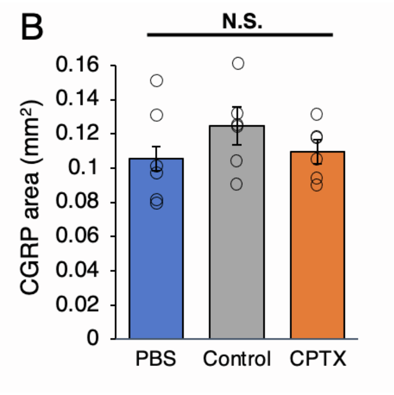

Figure S4. Immunohistochemical staining of calcitonin gene-related peptide (CGRP) (A) Representative images of CGRP-positive area at lumbar spinal cord. Scale bars: 1 mm.

(B) Quantitative analysis of CGRP-positive area of spinal dorsal horn in three groups(n=6 each PBS vs. control, $p = 0.3$, PBS vs. CPTX, $p = 0.13$, control vs CPTX, $p = 0.88$. Values are the mean ± SEM. Not significant (N.S.), $^*p < 0.05$, $^{**}p < 0.01$. Statistical analysis was performed using the Mann-Whitney U test following the Kruskal-Wallis test for CGRP analysis.

Supplemental Experimental Procedures

Lentiviral vector preparation

The construction data for the CPTX were provided by Dr. Suzuki (Keio University). The pLV-CAG-CPTX-His plasmid was generated using Vector Builder. Recombinant lentivirus expressing CAG-CPTX-His were generated through transient transfection with these plasmids: pCAG-HIVgp, pCMV-VSV-G-RSV-Rev (Miyoshi et al., 1998), and the lentiviral vector plasmid. Transfection of HEK293T cells was performed as previously described (Iida et al., 2017, Kojima et al., 2019). The culture supernatant containing the lentivirus was concentrated using ultracentrifugation (25000 rpm for 2 hours at 4 ℃). Viral particles were quantified by infecting HEK293T cells with serial dilutions of the vectors, followed by the measurement of the proportion of cells expressing the His-tag. The MOI for hiPSC-NS/PCs was calculated based on these functional titers.

Cell culture and lentiviral transduction

The integration-free human umbilical cord-derived hiPSC line YZWJs513, which was derived from a clinical-grade “human leukocyte antigen (HLA) superdonor” line (Umekage et al., 2019) at the Good Manufacturing Practice (GMP)-grade cell processing facility at the Center for iPS Cell Research and Application at Kyoto University (CiRA),

was used in this experiment. hiPSCs were produced in CiRA following previously reported the differentiation protocols for NS/PCs. Cells were cultured in a floating culture system. After culturing for 10 days, the cells were passaged, and infected with lentivirus. Lentivirus transduction was performed as previously reported (Nori et al., 2011;Okubo et al., 2018). Subsequently, the cells were cultured for an additional five days, and treated with N-[N-(3,5-difluorophenacetyl)-l-ananyl]-S-phenylglycine t-butyl ester (DAPT) (10μM, D5942, Sigma-Aldrich, St. Louis, MO, USA) for 1 day before transplantation, as described in a previous study (Sugai et al., 2021).

In vitro **and** ***in vivo*** **His-tag detection ELISA analysis**

Dissociated hiPSC-NS/PCs that had been infected with lentiviruses were seeded at a density of 3.5×10^5 cells/well in poly D-lysine/laminin-coated 24-well plate. After culturing for three days, the supernatant of the medium was collected, and the contents were determined using a His-tag ELISA Detection Kit (L00436, GenScript Biotech, USA). The average concentrations in the 4 wells were calculated.

Tissue samples were collected from rats at 13 weeks after transplantation (control, group: n = 4; CPTX group, n = 4). The entire brain of each rat was homogenized with a Radio-Immunoprecipitation Assay (RIPA) buffer and centrifuged to obtain a soluble fraction (15000 rpm for 30 minutes, 4 ℃). The supernatant was subjected to protein quantification using the Bradford method, and the protein concentration was adjusted to 1mg/ml. Venous blood serum was collected from the inferior vena cava prior to euthanasia. The samples were left at room temperature for 30 minutes, followed by centrifugation (3000 rpm for 15 minutes, 4 ℃) to remove blood clots. After clot removal, another centrifugation was performed (10000 rpm for 15 minutes, 4 ℃), and the supernatant was collected. Using the sample from the brain and venous serum, the His-tag concentration was measured using a His-tag ELISA Detection Kit.

PCR and electrophoresis for NRX SS4 detection

To confirm the expression of the CPTX-binding domain (NRX SS4) in hiPSC-NS/PCs, dissociated hiPSC-NS/PCs were cultured for 28 days in a serum-free medium in 12-well plates coated with poly-D-lysine/laminin. Total RNA was extracted from the differentiated transplanted cells using the RNeasy Micro Kit (Qiagen, Inc., Hilden, Germany), and cDNA was synthesized by reverse transcription using the ReverTra Ace qPCR RT master mix (Toyobo Co., Ltd., Life Science Department, Osaka, Japan). A mixture of cDNA and NRXSS4 manufactured primers (Applied FASMAC) was amplified by PCR. The PCR products were separated by electrophoresis on an 8% polyacrylamide gel. The primers

used for NRXSS4 in this study are as follows (5’-3’ orientation):
NRX1 (Forward): tcgccattgaagaatccaatg , (Reverse): ggttgcttggctattgaagat
NRX2 (Forward): gacgagcccaacgccatagtaa, (Reverse): tcttgatggcagcctggctgtt
NRX3 (Forward): caaagaggagagaacccctg, (Reverse): ggctatttgcgcctgagtgt

Cell viability and LDH release assay

The cell toxicity of the lentivirus was measured by using a Cell Counting Kit-8 (CK04; Dojindo, Kumamoto, Japan), and LDH release into the medium was measured using a Cytotoxicity LDH Assay Kit-WST (CK12; Dojindo) as previously described (Hayakawa et al., 2016). Briefly, hiPSC-NS/PCs were seeded in a 96-well plates at a density of 150000 cells per well. For the Cell Counting Kit-8 assay, lentivirus was added 48 h after cell suspension and incubated for 24 h. For the LDH assay, the lentivirus was added 48 h after cell suspension and incubated for 3 h. Then, cell viability and cell toxicity were calculated by reference to 450 nm and 490 nm light absorbance. Each simultaneously cultured well was considered as an individual sample for statistical analysis.

Animals

Adult (eight-week-old) female athymic nude rats (F344/NJcl-*rnu*/*rnu*, weight = 110–180 g, CLEA Japan, Inc., Tokyo, Japan) were used for these experiments. The rats were housed randomly in groups of three or four per cage (24 × 42 × 24 cm), regardless of the experimental group. They were kept on a 12/12 h light/dark cycle in an environment with controlled temperature and humidity and provided ad libitum access to food and water. Antibiotics (orbifloxacin; Sumitomo Dainippon Pharma Animal Health, Inc., Osaka, Japan) were administered for three days after SCI and other surgeries. All experimental procedures were approved by the Experimental Animal Care Committee of Keio University, School of Medicine (approval no. 13020) and were performed in accordance with the Guide for the Care and Use of Laboratory Animals (National Institutes of Health, Bethesda, MD). In this study, all rats were anesthetized by subcutaneous injection of 0.4 mg/kg medetomidine hydrochloride, 2 mg/kg midazolam, and 2.5 mg/kg butorphanol.

A total of 18 rats were used in each group for the in vivo experiments. The exclusion criteria were established as follows: 1. Rats displaying weight-supported walking (BBB score > 8) within 1 week after spinal cord injury, 2. Rats with an endpoint BBB score of ≥ 19, 3. Rats that developed severe pressure sores or soft tissue infections, 4. Rats among the transplanted group in which histological evaluation did not reveal HNA-positive cells (transplanted cells). The composition of each group was as follows: PBS

(weight-supported rats: n=2, infected rats: n=1), control (infected rats: n=1, BBB ≥ 19 rats: n=2, rats with no engrafted transplanted cells: n=2), CPTX (infected rats: n=2, rats without transplanted cells: n=1).

Surgical procedures

In this study, all rats were anesthetized by subcutaneous injection of 0.4 mg/kg medetomidine hydrochloride, 2 mg/kg midazolam, and 2.5 mg/kg butorphanol. Contusive SCI was induced at the level of the tenth thoracic spinal vertebra using an Infinite Horizon impactor (220 kdyn; Precision Systems and Instrumentation, Fair-fax Station, VA, USA) with a 2 mm tip, as described previously (Scheff et al., 2003). Nine days after the injury, hiPSC-NS/PCs ($1×10^6$ cells) were injected with a 27G metal needle using a micro stereotaxic injection system (KDS310; Muromachi-Kikai Co., Ltd.). An equal volume of PBS was similarly injected into PBS group rats. The injected depth was 0.4 to 1.0 mm and the injection speed was 1 μL/min. All experiments were performed in accordance with the Guidelines for the Care and Use of Laboratory Animals of Keio University (Tokyo, Japan, Permit Number; 13020) and The National Institutes of Health Guide for the Care and Use of Laboratory Animals. All surgeries were performed under anesthesia.

Histological analyses

Thirteen weeks after transplantation, the rats were anesthetized and euthanized by transcardial perfusion with glyoxal (Richter et al., 2018) followed by sequential soaking overnight in 10% and 30% sucrose. Spinal cord tissues were embedded in optimal cutting temperature (O.C.T.) compound (Sakura Finetechnical Co., Ltd., Tokyo, Japan) and sectioned at a thickness of 16 μm for the sagittal plane and 20 μm for the axial plane. Spinal cord sections were then immunohistochemically stained for histological analysis using the following primary antibodies: mouse anti-HNA: MAB4383, Merck Millipore, Burlington, MA, USA, 1:400), rabbit anti-MAP2 (AB5622, Merck Millipore, 1:500), mouse anti-ELAVL 3/4 (A21271, Molecular Probes Inc., Eugene, OR, USA, 1:100), rabbit anti-GFAP (16825-1-AP, Proteintech, Rosemont, IL, USA 1:2000), mouse anti-adenomatous poluposis coli CC-1 (APC: OP80, Merck Millipore, 1:400), rabbit anti-Nestin (18741, Immuno-Biological Laboratories, Gunma, Japan, 1:400), rabbit anit-Ki67 (NCL-Ki67p, Leica Biosystems, Richmond, IL, USA, 1:2000), mouse anti-synaptophysin (14-6525-82, Invitrogen, Waltham, MA, USA, 1:10000), anti-postsynaptic density 95 (PSD95: 51-6900, Thermo Fisher Scientific, Waltham, MA, USA, 1:100), mouse anti-human cytoplasm antibody (STEM121: Y40420, Cellartis-Takara Bio, Shiga, Japan, 1:200), goat anti-GFP (600-101-215, Rockland Immunochemicals, Pottstown, PA, USA, 1:1000), rabbit anti-

mCherry (167453, Abcam, Cambridge, UK, 1:500), mouse anti-vesicular glutamate transporter 2 (VGLUT2: S29-29, Thermo Fisher Scientific, 1:100), goat anti-vesicular GABA transporter (VGAT: Af620, Frontier institute, 1:250), rabbit anti-calcitonin gene-related peptide (CGRP: ab47027, Abcam, 1:400). Nuclei were stained with Hoechst 33258 (10 μg/ml). Spinal cord injury sites were stained with hematoxylin and eosin. Sample images were obtained using a fluorescence microscope (Leica Microsystem THUNDER imager Live Cell LAS X Version: 3.7.5.24914) or a confocal laser scanning microscope. Analyses were performed in a customized macro using ImageJ software (National Institute of Health, Bethesda, MD, USA).

G-deleted Rabies Virus Tracing Experiment

i) Generation of G-deleted rabies virus

The G-deleted rabies virus (RABVΔG) was generated using a method similar to that previously reported (Osakada et al., 2013). The genomic plasmids required for the virus creation,namely pSADΔG-mCherry, pcDNA-B19, pcDNA-B19L, pcDNA-B19G (available from addgene), and B7GG cells, and BHK-EnvA cells were kindly provided by Dr.F.Osakada (Nagoya University, Nagoya, Japan).The following steps were used to create the RABVΔG. 1. Reconstruction of RABVΔG from the provided cDNA. 2. Introduction of genes using pSADΔG-mCherry, pcDNA-B19, pcDNA-B19L, pcDNA-B19G into B7GG cells and collected the supernatant. 3. Amplifying the reconstructed RABVΔG in B7GG cells. 4. Infection of BHK-EnvA cells with the supernatant from step 3 to perform pseudotyping with EnvA. 5. Concentrate the virus by subjecting the supernatant from step 4 to two rounds of ultracentrifugation. 6. Measure the titer of EnvA-RABVΔG using HEK-TVA cells. These processes followed the protocol described in Osakada et al., 2013, with specific conditions and materials as mentioned in the same reference.

ii) Lentivirus production for TVA and G protein gene delivery

A plasmid named pBOB-synP-HTB (available from addgene) contains TVA receptor and G protein expression was provided by Dr. Osakada. The gene construct includes hSyp-EGFP-2A-TVA-2A-RBG. To generate the recombinant lentivirus, this plasmid was transfected into HEK293 cells together with other plasmids, pCAG-HIVgp and pCMV-VSV-G-RSV-Rev. Transfection of HEK293T cells was carried out as previously described (Iida et al., 2017, Kojima et al., 2019).The culture supernatant containing the lentivirus was concentrated through ultracentrifugation (25000 rpm for 2 hours at 4°C). Viral particles were quantified by infecting HEK293T cells with serial dilutions of the vectors, followed by measuring the proportion of cells expressing EGFP. The Multiplicity of

Infection (MOI) for hiPSC-NS/PCs was calculated based on these functional titers.

iii) Virus infection to hiPSC-NS/PCs and administration

Lentivirus containing hSyn-TVA-RVG was administered to hiPSC-NS/PCs four days prior to transplantation. Specifically, in the CPTX group, the medium was changed the following day, and the lentivirus inducing CPTX was administered. After transplantation of these cells (1×10^6 cells), 12 weeks later, G-deleted rabies virus (titer: 1.8×10^8 vg/ml) was injected bilaterally at the transplantation site (2-point injection, 750 nl per injection, located 500 μm laterally from the center of the spinal cord and at a depth of 0.4–1mm from the surface). A glass syringe was used for the injections, and specific post-injection procedures, waiting periods, and needle removal methods were used to prevent viral leakage during administration. Immunohistochemical staining of cervical sections was performed to evaluate the tracts seven days after the injections.

Quantification of staining

Immunohistochemical staining of all sections was quantified using ImageJ software. Threshold values were maintained consistently across all analyses by observers who were blinded to the experimental conditions and groups. Human human-specific synaptophysin and VGLUT2 were quantified by capturing images at 20x magnification at the center of the transplanted region and tiling them to assess the area across all axial section fields. To quantify PSD95, we randomly selected one field at 63x magnification from the central transplantation region (two areas per slide) and measured the area. In the CPTX group, the inclusion of His-tag-positive cells within the field was required for analysis. To assess synaptic formation, the contact points between the presynaptic and postsynaptic markers were indirectly defined as synapses (Suzuki et al., 2020). Using ImageJ software, the overlapping areas of synaptophysin and PSD95 were extracted, and the number of these puncta was quantified. To account for bias, the number of synaptophysin puncta was normalized. The location of RST in the G-deleted rabies virus tracing experiment was verified based on previous studies (Liang et al., 2017). The calculation was based on the average area of the RST on both sides.

Motor-evoked potential experiments

Electrophysiological experiments were performed using a Neuropack S1 MEB9402 signal processor 98 days after transplantation (n = 7 each), as described previously (Nori et al., 2011). The surface of the T3 spinal cord was stimulated, and needle electrodes were used to record the signal from the hind limb. An active electrode was placed in the quadriceps muscle, a reference electrode was placed near the distal quadriceps muscle

tendon, and the ground electrode was placed on the back muscle. The stimulus parameters were an intensity of 3 mA, a duration of 0.2 ms, and an interstimulus interval of 1 Hz.

Statistical analyses

Statistical analyses were performed using SPSS (Japan IBM, Tokyo, Japan, Ver. 26.0.0.0).The normality of the distribution of data points was verified using Shapiro-Wilk test. Data are reported as the mean ± standard error of mean (SEM). Sample sizes are indicated in the respective figure legends. The Mann-Whitney U-test was used for comparisons between two groups *in vitro* and *in vivo* immunohistochemistry (IHC) staining results. All multiple testing data were analyzed by the Kruskal-Wallis test in treadmill gait, muscle weight, CGRP staining, allodynia and MEP assays. Two-way repeated measures analysis of variance (ANOVA) was used for weekly BBB scoring. P values < 0.05 indicate statistical significance (as * $p < 0.05$, ** $p < 0.01$).

Supplemental References

1. Iida, T., Iwanami, A., Sanosaka, T., Kohyama, J., Miyoshi, H., Nagoshi, N., Kashiwagi, R., Toyama, Y., Matsumoto, M., Nakamura, M., and Okano, H. (2017). Whole-Genome DNA Methylation Analyses Revealed Epigenetic Instability in Tumorigenic Human iPS Cell-Derived Neural Stem/Progenitor Cells. Stem Cells *35*, 1316-1327. 10.1002/stem.2581.
2. Kojima, K., Miyoshi, H., Nagoshi, N., Kohyama, J., Itakura, G., Kawabata, S., Ozaki, M., Iida, T., Sugai, K., Ito, S., et al. (2019). Selective Ablation of Tumorigenic Cells Following Human Induced Pluripotent Stem Cell-Derived Neural Stem/Progenitor Cell Transplantation in Spinal Cord Injury. Stem Cells Transl Med *8*, 260-270. 10.1002/sctm.18-0096.
3. Liang, H., Paxinos, G., and Watson, C. (2012). The red nucleus and the rubrospinal projection in the mouse. Brain Structure and Function *217*, 221-232. 10.1007/s00429-011-0348-3.
4. Miyoshi H, Blomer U, Takahashi M, Gage FH, Verma IM. Development of a self-inactivating lentivirus vector. J Virol. 1998;(10):8150-7
5. Nori, S., Okada, Y., Yasuda, A., Tsuji, O., Takahashi, Y., Kobayashi, Y., Fujiyoshi, K., Koike, M., Uchiyama, Y., Ikeda, E., et al. (2011). Grafted human-induced pluripotent stem-cell-derived neurospheres promote motor functional recovery after spinal cord injury in mice. Proc Natl Acad Sci U S A *108*, 16825-16830. 10.1073/pnas.1108077108.

6. Okubo, T., Nagoshi, N., Kohyama, J., Tsuji, O., Shinozaki, M., Shi- bata, S., Kase, Y., Matsumoto, M., Nakamura, M., and Okano, H. (2018). Treatment with a gamma-secretase inhibitor promotes functional recovery in human ipsc- derived transplants for chronic spinal cord injury. Stem Cell Rep. 11, 1416–1432.
7. Richter, K.N., Revelo, N.H., Seitz, K.J., Helm, M.S., Sarkar, D., Saleeb, R.S., D'Este, E., Eberle, J., Wagner, E., Vogl, C., et al. (2018). Glyoxal as an alternative fixative to formaldehyde in immunostaining and super-resolution microscopy. EMBO J *37*, 139-159. 10.15252/embj.201695709.
8. Umekage, M., Sato, Y., and Takasu, N. (2019). Overview: an iPS cell stock at CiRA. Inflamm Regen *39*, 17. 10.1186/s41232-019-0106-0.
9. Scheff, S.W., Rabchevsky, A.G., Fugaccia, I., Main, J.A., and Lumpp, J.E., Jr. (2003). Experimental modeling of spinal cord injury: characterization of a force-defined injury device. J Neurotrauma *20*, 179-193. 10.1089/08977150360547099.
10. Sugai, K., Sumida, M., Shofuda, T., Yamaguchi, R., Tamura, T., Kohzuki, T., Abe, T., Shibata, R., Kamata, Y., Ito, S., et al. (2021). First-in-human clinical trial of transplantation of iPSC-derived NS/PCs in subacute complete spinal cord injury: Study protocol. Regen Ther *18*, 321-333. 10.1016/j.reth.2021.08.005.
11. Suzuki, K., Elegheert, J., Song, I., Sasakura, H., Senkov, O., Matsuda, K., Kakegawa, W., Clayton, A.J., Chang, V.T., Ferrer-Ferrer, M., et al. (2020). A synthetic synaptic organizer protein restores glutamatergic neuronal circuits. Science *369*. 10.1126/science.abb4853.
